# QRS Detector Performance Evaluation Aware of Temporal Accuracy and Presence of Noise

**DOI:** 10.3390/s24051698

**Published:** 2024-03-06

**Authors:** Wojciech Reklewski, Marek Miśkowicz, Piotr Augustyniak

**Affiliations:** Department of Metrology and Electronics, Biocybernetics ad Biomedical Engineering, AGH University of Krakow, 30-059 Krakow, Poland; reklewsk@agh.edu.pl (W.R.); miskow@agh.edu.pl (M.M.)

**Keywords:** electrocardiogram (ECG), QRS detection, ECG interpretive software testing, biomedical signal processing, MIT-BIH Arrhythmia Database

## Abstract

Algorithms for QRS detection are fundamental in the ECG interpretive processing chain. They must meet several challenges, such as high reliability, high temporal accuracy, high immunity to noise, and low computational complexity. Unfortunately, the accuracy expressed by missed or redundant events statistics is often the only parameter used to evaluate the detector’s performance. In this paper, we first notice that statistics of true positive detections rely on researchers’ arbitrary selection of time tolerance between QRS detector output and the database reference. Next, we propose a multidimensional algorithm evaluation method and present its use on four example QRS detectors. The dimensions are (a) influence of detection temporal tolerance, tested for values between 8.33 and 164 ms; (b) noise immunity, tested with an ECG signal with an added muscular noise pattern and signal-to-noise ratio to the effect of “no added noise”, 15, 7, 3 dB; and (c) influence of QRS morphology, tested on the six most frequently represented morphology types in the MIT-BIH Arrhythmia Database. The multidimensional evaluation, as proposed in this paper, allows an in-depth comparison of QRS detection algorithms removing the limitations of existing one-dimensional methods. The method enables the assessment of the QRS detection algorithms according to the medical device application area and corresponding requirements of temporal accuracy, immunity to noise, and QRS morphology types. The analysis shows also that, for some algorithms, adding muscular noise to the ECG signal improves algorithm accuracy results.

## 1. Introduction

QRS detectors are widely used as a front end of various ECG processing chains; consequently, their usage is concerned virtually in any ECG-dedicated software. Moreover, from the signal processing viewpoint, they serve as an interface between a continuous series of samples captured by recording devices and a feature-based algorithm used for classification and detection purposes. Consequently, in several ECG software packages, the QRS detector and preceding filters are the unique procedures involving every sample of the record. Therefore, high accuracy of detection and low computational complexity are of key importance and are usually regarded as primary quality factors. Detection accuracy, however, becomes a less distinctive factor as many modern detectors approach 99.9%.

Mobile and wearable ECG data acquisition systems face an inherent conflict between autonomy time and usability. The time between battery recharge, power consumption, detection accuracy, size, and weight of the device have to be balanced by a compromise. To this end, two important quality aspects appear to produce a reliable estimate of the suitability of a particular QRS detection algorithm to an area of application. The first aspect is the temporal stability of the detection point sequence (that is, its independence from the QRS morphology type). With the QRS detector producing highly accurate detection points, one could avoid recalculations of more precise QRS peak positions and directly input the detection points to procedures such as HRV analysis or shape classifying. The second point is the noise immunity of the detection point sequence, which specifies how much noise (e.g., of muscular origin), present for example in wearable-based in-field ECG measurements, affect the precision of QRS detection and alter the medical findings produced by subsequent diagnostic procedures.

In this paper, we address the problem of the comprehensive evaluation of QRS detection performance using multidimensional criteria, including the temporal tolerance of QRS complex detection, robustness amid noise, and sensitivity to QRS morphology. The proposed methodology enables the selection of the QRS detectors for specific applications, such as clinical data analysis, long-term monitoring with mobile devices and wearables, fast results, or arrhythmia diagnosis. The analysis is demonstrated in the example of four selected QRS detection algorithms implemented in Python according to their specification in the relevant references [1,2,3,4]. The algorithms are tested on the MIT-BIH Arrhythmia Database (MIT-BIH AD) [5] with added muscular noise from the MIT-BIH Noise Stress Test Database (MIT-BIH NSTD) [6]. The tests were conducted on Dell Latitude E6400, Intel Core2Duo P8400, 2.26 GHz, and 4 GB RAM running with Debian 10.13. Implementation of the algorithms, test tools, and data processing were performed in Python 3.7.3. Plots were created in Jupyter Notebook (server v5.7.8 with Python 3.7.3 [GCC 8.3.0]).

The performance analysis shows that the QRS detectors demonstrate different sensitivities to the detection of temporal tolerance. Some algorithms maintain high detection accuracy, even for low values of temporal tolerance of QRS detection. The others exhibit good results only for high values of temporal tolerance (i.e., comparable to the QRS standard duration of 100 ms).

The QRS detection performance under extra noise is in general deteriorated. However, as shown in this paper, for some QRS morphologies, extra noise in the ECG signal can paradoxically improve QRS detection accuracy. This effect resembles the improvement of audio and video data by randomizing the quantization error known as dither [7,8].

The performance of the algorithms depends on QRS morphology, as each algorithm uses different ECG signal filtering methods and signal analysis in subsequent processing blocks. Some QRS morphologies are more problematic than others for each algorithm under analysis. In our tests, V-type QRS morphologies were the most problematic for algorithms under analysis.

The paper is organized as follows: Section 2 addresses related work; Section 3 describes the four algorithms under analysis, the database, and the preparation of test datasets; Section 4 presents results; Section 5 is the discussion; and Section 6 contains conclusions.

## 2. Related Work

Medical testing procedures are conventionally evaluated based on binary classification by calculating parameters such as TP, FN, FP, and TB. These parameters are also commonly used in the literature for performance evaluation and comparison of QRS detection algorithms [9]. True positive (TP) is the number of correctly detected R peaks, false negative (FN) is the number of omitted R-peaks, false positive (FP) is the number of places wrongly classified as R-peaks, and total beats (TB) is the number of annotated R-peaks in a database record. However, much less attention is paid to temporal detection accuracy. The numerical values of TP, FN, and FP depend on detector temporal tolerance (DTT), defined as the maximum allowed time difference between the algorithm detection points (R-peaks) and the corresponding annotations from the reference database [10]. A variety of temporal tolerance values are used in the literature, ranging from 60 ms to 160 ms, which sometimes results in the comparison of algorithms with different temporal resolutions. The problem of the sensitivity of the accuracy of QRS detection algorithms in the temporal resolution of the detection defined by DTT is examined in [10]. When the algorithm is tested with a certain high value of DTT, relatively distant locations of R-peak detection points and database annotation are successfully paired and counted as TP. Further testing with certain lower DTT values will result in the distance between detection points and database annotation exceeding the DTT value and, consequently, their pairing will be unsuccessful. Database annotation without paired detection points will be counted as FN, and algorithm detection without paired database annotation will be counted as FP. This way, the algorithm’s TP detection for higher values of DTT will be replaced by a pair of FN and FP detections for lower DTT values. As expected, the higher the DTT, the better the numerical results of the TP, FN, and FP. However, the deterioration rate of an algorithm’s accuracy depends on the given QRS detection algorithm. Some algorithms demonstrate slow degradation, while others suffer a quick performance drop with decreasing DTT values [10].

A substantial research effort has been dedicated to examining the robustness of QRS detectors’ performance against noise in ECG signal recordings; [11] reviews 38 major state-of-the-art techniques of QRS detection with comprehensive comparative analysis of techniques for ECG signal denoising and QRS detection. In their conclusion, the authors emphasize a need to invent computational techniques “to analyze the ECG signal with higher accuracy in all conditions”, which justifies the development of multidimensional methods to test algorithms’ performance, among other criteria, in various noise conditions and temporal accuracy requirements.

The performance results of the Pan–Tompkins QRS detection algorithm in noisy ambulatory ECG data with varying signal-to-noise ratios are presented in [12]. Two ECG databases are used for testing: the MIT-BIH NSTD [6] and the MIT-BIH AD [5]. The algorithm results for sensitivity (*Se = TP*/(*TP + FN*)) and positive predictivity (*PPV* or *+P*) (*+P = TP*/(*TP + FP*)) deteriorate from close to 100% for noise level with SNR = 24 dB to around 60% and 70% respectively for SNR = −6 dB. The analysis shows that the Pan–Tompkins algorithm needs improvements to achieve good detection performance for noisy signals.

In [13], the performance analysis of selected three well-known QRS detection algorithms is addressed: by Pan–Tompkins [4], WQRS [14], and by Hamilton [15] against the MIT-BIH AD and the noise-contaminated ECG signal with different levels of baseline wander (BW), muscle artifact (MA), and electrode motion (EM) artifact from the MIT-BIH NSTD. As shown in [13], noise and artifacts decreased the quality indices of algorithms from close to 100% for SNR = 12 dB to:

- BW noise: Se = 95% for WQRS and +P = 62% for WQRS for SNR = −12 dB,
- MA noise: Se = 83% for Hamilton and +P = 38% for WQRS for SNR = −12 dB,
- and EM noise: Se = 65% for Hamilton and +P = 30% for WQRS for SNR = −12 dB.

The poorest performance was noted for ECG signals affected by EM artifacts.

In [16], a new QRS detection method is proposed and validated, with different levels of baseline wander, muscle artifact, and electrode motion artifact as noise sources against MIT-BIH NSTD with the following processing blocks: first derivative, Hilbert transform envelope, wavelet transform, wavelet component reduction, signal reconstruction, and thresholding. The proposed QRS detection method achieves Se = 78.89% and +P = 75.25% for MIT-BIH NSTD and SNR = 0 dB.

The performance of three selected state-of-the-art QRS detection algorithms and the evaluation of the accuracy of their R-peak localization are included in [17]. The algorithms under analysis were the following: integrate and fire pulse train automaton [18], zero-crossing counts [19], and the knowledge-based method [20]. The authors propose a method to estimate the temporal accuracy of R-peak detection for normal and abnormal beats as well as a simple scheme to compensate for slackness introduced by the filtering part of the algorithms.

In opposition to the ubiquitous, conventional, TP, FN, and FP parameters mentioned at the beginning of this section, a novel QRS detection performance indicator, jitter with accuracy (JA), aimed at evaluating QRS detection algorithms under realistic noise scenarios, is proposed in [21]. The authors state that *Se* and +P metrics used to assess the quality of R-peak detection lose information value, where a high temporal tolerance of 100 ms or more [21] is used. Also, frequent use of the MIT-BIH AD, which is, according to the authors, artifact-free, leads to an overestimation of algorithm performance and unjustified reported *Se* and *+P* performance indicators significantly above 99%.

The relationship between QRS detection performance and database sampling frequency is examined in [22]. The analysis, carried out for the Hamilton algorithm [23] against the MIT-BIH database, shows that adapting the algorithm threshold parameters to sampling frequency optimizes the algorithm’s accuracy results.

In [24], the five selected multisignal heartbeat detectors are tested against 100 records from the training dataset of the PhysioNet/CinC Challenge 2014, with various noise levels added. The performance results and best-worse ranking of the detectors are reported.

The performance of 10 QRS detection algorithms against six internationally recognized ECG databases with various normal and abnormal beat types and various levels of noise and artifacts is reported in [25]. The tested algorithms were by Pan–Tompkins, Hamilton mean, Hamilton median, RS slope, sixth power, finite state machine (FSM), U3 transform, difference operation (DOM), ‘jqrs’, and optimized knowledge based (OKB). The overall results are reported for each algorithm and each database. The analysis shows that QRS detection results decrease significantly for poor signal-quality ECG signals for all tested algorithms.

A new R-peak detector based on neural networks is proposed in [26]. The algorithm performance does not deteriorate with low-quality or noisy ECG signals acquired from mobile electrocardiogram sensors, such as Holter monitors. The proposed QRS detector uses a 1-D self-organized operational neural network with generative neurons and offers lower computational complexity than conventional 1-D convolutional neural networks. The QRS detection results reported are a 99.10% F1 score (F1 = 2 · PPV · Se/(Se + PPV)), 99.79% *Se*, and 98.42% *+P* achieved on the China Physiological Signal Challenge-2020 dataset (CPSC-DB). The CPSC-DB database contains 1 026 095 beats, collected from arrhythmia patients, and includes real-world noise as well as artifacts from a wearable real-world Holter ECG device.

In [27], 10 QRS detection techniques published between 2020 and 2022 are compared based on the performance parameters: Se, PPV, F1 score, and DER (DER = (FN + FP)/TB).

A new R-peak detection technique based on visibility graph transformation, which maps a discrete time series to a graph by expressing each sample as a node and assigning edges between intervisible samples, is proposed in [28]. The proposed method is compared against two existing QRS detection methods on a noisy and sample-accurate University of Glasgow ECG Database [29] with two performance metrics: F1 score and root mean square of successive differences (RMSSD). The result of the first comparison is presented as a boxplot of the proposed F1 score method versus the SWT-based method [30] and matched filter detector [21]. There is an annotation that “the tolerance for deviation from the true R-peaks was 0%”, which corresponds to DTT = 0. In order to evaluate the RMSSD, a Wilcoxon signed-rank test between the estimated RMSSD values and the ground truth was performed and presented.

In [31], the authors present two (FastNVG and FastWHVG) computationally accelerated versions of the visibility graph transformation QRS detection method proposed in [28], together with an extended comparison with additional 7 QRS detectors from the literature.

ECG noise removal techniques are reviewed in [32]. The types and sources of noise are identified, and six major domains of denoising are subsequently explored. The techniques for denoising are presented and their performance is evaluated according to the following parameters: root-mean-square error, percentage-root-mean-square difference, and signal-to-noise ratio improvement.

## 3. Materials and Methods

To demonstrate the concept of evaluation of QRS detector performance aware of temporal accuracy, the presence of noise, and various QRS morphologies, we implemented four QRS detection algorithms and tested their accuracy, expressed by a true-positive-to-total-beats ratio (TP/TB). The tests are carried out for a range of DTT values and controlled mixing of muscular noise, which is often present in wearables applications. Obviously, it is desirable for QRS detectors to show good TP/TB scores, even for low DTT. Such detectors are particularly welcome, as R-peak location corrections are not necessary, and resynchronization of the heartbeat time series is not needed before further ECG processing steps.

The four algorithms selected from the QRS detection literature consist of three algorithms developed for mobile and wearables applications [1,2,3] and the algorithm broadly used as the reference in QRS detection literature [4]. Algorithms 1 and 2 belong to a group of algorithms based on digital filtering [9]. In addition, they can be classified into a group of “low” computational complexity using the subjective comparison with respect to computational load [9]. In contrast, Algorithm 3 does not use any digital filters and works on a different principle of operation based on a level-crossing sampling of the ECG signal. Algorithm 3 can be assigned to the group of “medium” computational load. Algorithm 4 is based on digital filters [9] and can be assigned to the group of “medium” computational load. Compared with Algorithms 1 and 2, Algorithm 4 uses more mathematical operations performed in multiple stages of signal processing.

Section 3.1, Section 3.2, Section 3.3 and Section 3.4 present the specification of QRS detection algorithms included in the study, while Section 3.5 contains the definition of test signals used to examine their immunity to noise in ECG signals.

### 3.1. Algorithm 1

**Figure 1:**
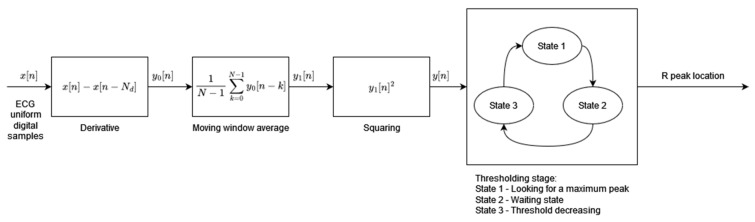
Algorithm 1 block diagram based on [1].

Algorithm 1 has been designed to address the requirements of low-power and real-time operations for use in mobile and wearable applications. The algorithm’s two main processing blocks, preprocessing and dynamic thresholding, have been designed to minimize necessary computational resources and power consumption (Figure 1). The preprocessing block consists of three consecutive operations performed on the input digital ECG signal: differentiation, the moving window average, and squaring. The resultant feature signal is fed to the thresholding block, which works in sequence in three states. In State 1, the algorithm searches for the maximum value of the feature signal within a window of fixed length (260 ms). The time instant when the feature signal reaches its maximum is marked as an R-peak temporal location. State 2, following State 1, is a 200 ms wait from the R-peak detection. During State 3, the dynamic threshold is decreased exponentially with time until it reaches the value of the feature signal. State 3 is then terminated and the algorithm moves to State 1, the search for the maximum value of the feature signal. The threshold initial value in State 3 is adaptive and dependent on the average amplitude of all previously found R-peaks.

### 3.2. Algorithm 2

**Figure 2:**
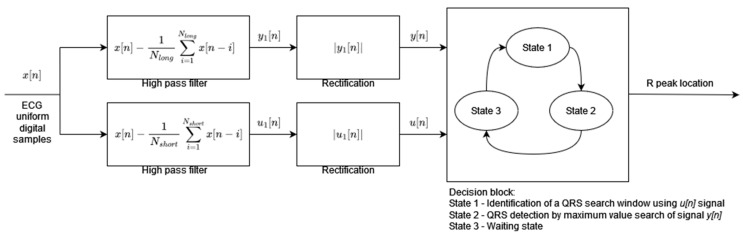
Algorithm 2 block diagram based on [2].

Algorithm 2 has been designed to achieve the low computational complexity and high energy efficiency needed for mobile and portable applications. The preprocessing stage consists of two parallel signal processing paths (Figure 2). Both paths include the high-pass moving average filters but with different cutoff frequencies followed by a rectification operation. The moving average window lengths *N_long_* and *N_shor_*_t_ define the cutoff frequencies of the filters. The outputs of the parallel processing paths are the inputs to the decision block. The preprocessed signal *u*[*n*], after high-pass filtering with a higher cutoff frequency (moving average window length *N_short_*), is used to decide when to start the QRS search window. During the search window of a fixed 200 ms length, the preprocessed signal of the other path *y*[*n*] after high-pass filtering with a lower cutoff frequency (moving average window length *N_Long_*) is analyzed in order to find its maximum value. The time instant of the maximum value of the *y*[*n*] signal is classified as an R-peak occurrence. The decision block of the algorithm works in three states: (1) identification of the search window, (2) detection of the R-peak by maximum *y*[*n*] value search, and (3) waiting state after R-peak occurrence. The threshold necessary to identify the search window is adaptive and its value is calculated based on the amplitude of the last R-peak and the previous threshold value.

### 3.3. Algorithm 3

**Figure 3:**
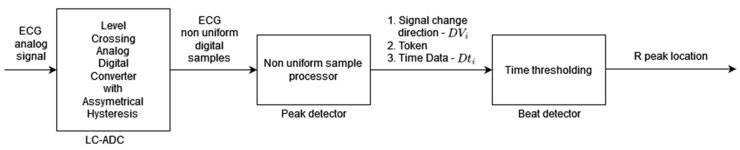
Algorithm 3 block diagram based on [3]. Peak detector output signal *DV_i_* is a two-bit signal where values 00 or 11 indicate the local peak in the ECG signal; that is, the sample that is taken when the input signal crosses *k_l_* sampling levels in the opposite direction to the sample taken previously. *Token* is a one-bit signal indicating with “1” the moment of sampling, and *Dt_i_* is an 11-bit word readout of the counter to register information about sample time (required in non-uniform sampling).

Algorithm 3 [3] is based on modified level-crossing sampling, which belongs to event-triggered sampling schemes [33]. The input to the algorithm is the analog ECG signal (instead of the digital ECG used for the other algorithms analyzed in this paper). In the level-crossing sampling, the analog input range is divided into 2^M^ − 1 levels (where M is the sampling resolution) and the sample is taken only when the input signal crosses one of the levels. The level-crossing analog-to-digital converter (LC-ADC) used in this algorithm is modified by asymmetrical hysteresis. The sample is taken only when (a) the input signal crosses the sampling level in the same direction as the last sample taken or (b) the input signal crosses *k_l_* levels in the opposite direction (where *k_l_* is the hysteresis parameter). The LC-ADC outputs the samples that are non-uniformly spaced in time. By selecting the appropriate value of *k_l_*, analysis of sample clusters (Figure 3 signal change direction *DV_i_*, *Token*)*,* and their timing (Figure 3 Time Data *Dt_i_*), it is possible identify the input ECG signal peaks first and, in the next processing step, identify R-peaks. There are three main processing blocks: level-crossing analog-to-digital converter (LC-ADC), peak detector, and beat detector (Figure 3). The algorithm does not use any filters (unlike other algorithms analyzed in this paper) due to the properties of the level-crossing sampling scheme adopted in the LC-ADC with hysteresis. For the purposes of algorithm analysis in this paper, the input to the algorithm is a uniformly sampled ECG signal.

### 3.4. Algorithm 4

**Figure 4:**
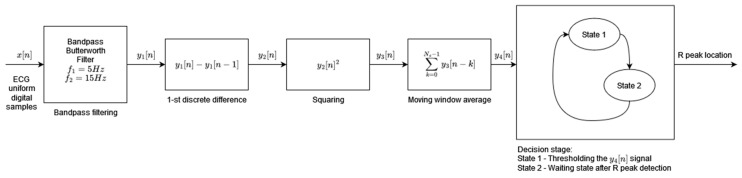
Algorithm 4 block diagram based on [4].

Algorithm 4, developed by Pan and Tompkins and published in 1985, is the most widely referenced QRS detector. The adaptation of the original algorithm used in this study comes from [34]. In this adaptation, the decision block is simplified, whereas the preprocessing stage uses the Butterworth filter instead of the simple moving average filter. The original ECG signal preprocessing is retained and consists of a band pass filtering, differentiation, squaring, and moving window average [4] (Figure 4). The resulting preprocessed signal is fed to the decision block where, during State 1, its first local maximum that is higher than the detection threshold is marked as the R-peak (the condition for a local maximum is where the next and the previous sample values are smaller than the sample under analysis). Each local maximum smaller than the detection threshold modifies the value of the detection threshold in line with [4]. As soon as the R-peak is found, the algorithm enters the waiting state, State 2, which is 200 ms long, and thereafter resumes the local maximum search. The parallel analysis of filtered signals with a second threshold is not applied in this implementation of the original algorithm. The modification of the feature signal threshold based on a regular and irregular heart rate, as well as the searchback mechanism, is implemented.

### 3.5. ECG Database and Test Dataset

In the MIT-BIH AD, the QRS morphology types [35] appear with the following number of cases: N (Normal)—75052, L (Left Bundle Branch Block)—8075, R (Right Bundle Branch Block)—7259, V (Ventricular Premature Beat)—7130, P (Paced)—7028, and A (Atrial Premature Beat)—2546. These six QRS morphology patterns were selected as the most frequent in the database (107090/109494 beats, i.e., 97.8%) as well as in real recordings expected in mobile patients. Records in the MIT-BIH AD are sampled at 360 Hz, which corresponds to the sampling interval of 2.7778 ms. Throughout this paper, we use the number of samples to describe the timeline.

#### Noise Pattern and Noisy Test Signals

In order to evaluate the immunity to noise of the QRS detectors under analysis, we have used the first channel signal from the MIT-BIH AD and three derivate datasets. Each of the three datasets was created by adding a noise signal from the MIT-BIH NSTD multiplied by three different scaling factors to the original MIT-BIH AD. Considering the wearable application as the most expected and the omnipresence of muscle artifacts, we decided to use a “muscle artifact” (MA) record from the MIT-BIH NSTD. From a practical viewpoint, the records were made with the same sampling parameters and the same length as the ECG signal. Moreover, the noise added is point-by-point trackable, allowing for a detailed comparison of the detector’s performance at each particular heartbeat. As we used original records from the MIT-BIH AD, the intrinsic noise already present in the data is out of our control. Consequently, the investigation of QRS detectors’ behavior in the presence of noise refers to “original” and not “noise-free” ECGs, and the relative signal-to-noise ratio (SNR) has been calculated based on the average power factor of the original record *P_s_* (MIT-BIH AD) and added noise pattern *P_n_* (MIT-BIH NSD record MA):

(1) $$SNR=20log\frac{P_{s}}{P_{n}}$$

The power is calculated according to:

(2) $$P=\frac{1}{N-1}\sum_{i=0}^{N-1}{\left(x_{i+1}-x_{i}\right)}^{2}$$

The following procedure has been applied to achieve the target relative SNR of 15, 7, and 3 dB. The mixing procedure for one record of the MIT-BIH AD starts with the calculation of SNR based on Equation (1), where *P_s_* is the power factor for this MIT-BIH AD record and *P_n_* is the power factor for the MIT-BIH NSTD record MA. The result is existing SNR (eSNR). Next, to calculate *k* (scaling factor), the target noise level (tSNR; for example, 3 dB) is input to Equation (3). The square root in Equation (3) is due to the fact that the amplitude *ECG_test_* and *ECG_orig_* ratio is the square root of the power ratio. Once the scaling factor *k* is calculated, the test dataset *ECG_test_* is calculated according to Equation (4), where *MA* is the MIT-BIH NSTD record MA. This procedure is repeated for all MIT-BIH AD records for all three (15, 7, 3 dB) noise-test datasets.


(3) $$k=\sqrt{\frac{tSNR}{eSNR}}$$

(4) $$ECG_{test}=ECG_{orig}+k\cdot MA\;$$

**Figure 5:**
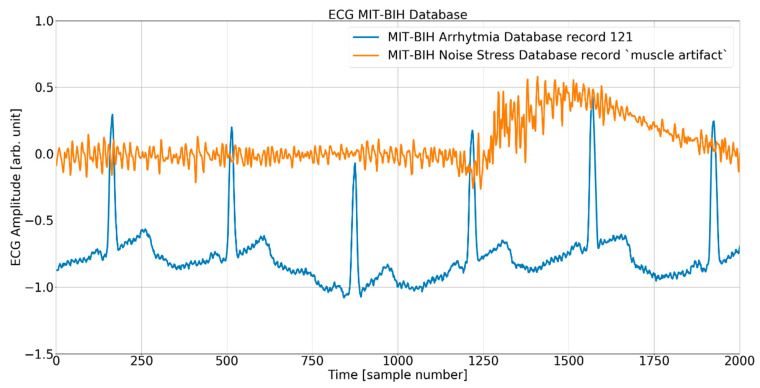
The ECG (excerpt of record 121 from the MIT-BIH AD) and noise (excerpt of record MA (muscle artifact) from the MIT-BIH NSTD) before the mixing procedure.

**Figure 6:**
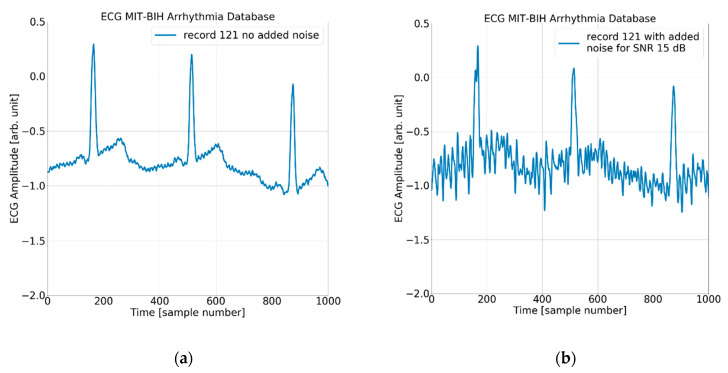

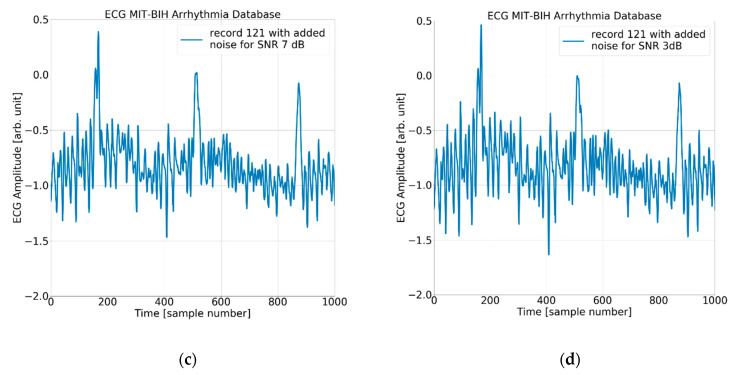
The ECG (excerpt of record 121 from the MIT-BIH AD) with (**a**) no added noise, (**b**) added noise for SNR = 15 dB, (**c**) added noise for SNR = 7 dB, and (**d**) added noise for SNR = 3 dB.

The process of adding noise to example record 121 of the MIT-BIH AD is illustrated in Figure 5 and Figure 6 below.

## 4. Results

**Figure 7:**
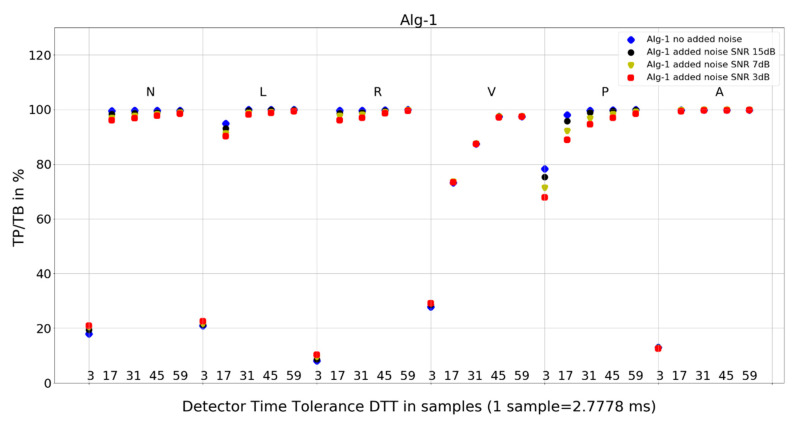
Performance of Algorithm 1 in relation to QRS beat morphology (upper long horizontal axis), DTT values (in samples, bottom short horizontal axes), and added noise level (dot shape and color).

**Figure 8:**
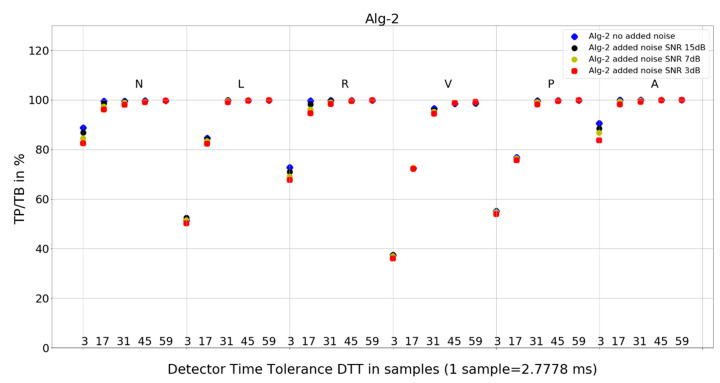
Performance of Algorithm 2 in relation to QRS beat morphology (upper long horizontal axis), DTT values (in samples, bottom short horizontal axes), and added noise level (dot shape and color).

**Figure 9:**
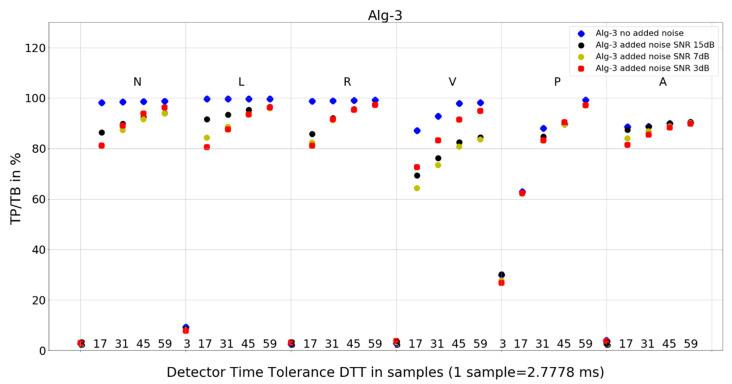
Performance of Algorithm 3 in relation to QRS beat morphology (upper long horizontal axis), DTT values (in samples, bottom short horizontal axes), and added noise level (dot shape and color).

**Figure 10:**
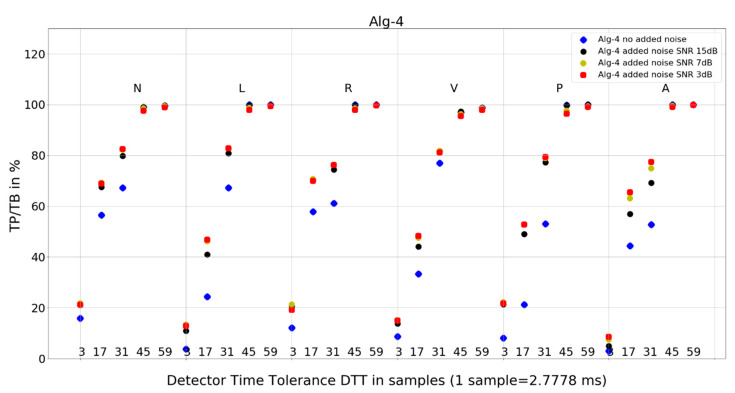
Performance of Algorithm 4 in relation to QRS beat morphology (upper long horizontal axis), DTT values (in samples, bottom short horizontal axes), and added noise level (dot shape and color).

The investigation of the QRS detection accuracy of four QRS detectors was performed separately for the six most frequent beat morphologies, five arbitrarily selected values of detection temporal tolerance, and four true-to-life levels of noise mixed with the database records. The amount of data to analyze and present is sizeable. Therefore, we present the following:

- statistics of the results in Table 1, Table 2 and Table 3, and the mean and standard deviation of TP/TB,
- the plot for each algorithm in Figure 7, Figure 8, Figure 9 and Figure 10, presenting 120 data points for each algorithm, with data points calculated from totals of detailed results,
- and 8 tables from 120 tables, with detailed results for individual records and totals for a given set of DTT, four noise levels, and QRS morphology (Table 4, Table 5, Table 6, Table 7, Table 8, Table 9, Table 10 and Table 11).

### 4.1. Statistics of the QRS Detectors’ Performance

With four independent variables (detector number, DTT, QRS morphology type, and added noise level), one can build a total of 16 statistical analyses. We selected the three most representative statistical analyses for independent studies of the detectors’ performance and vulnerability to DTT, QRS morphology type, and added noise level. Table 1 summarizes statistics on TP/TB for each algorithm (columns) and each tolerance window length (rows) for various DTT values, while the mean value and standard deviation are calculated for all QRS morphology types and added noise levels.

Table 2 presents statistics on TP/TB for each QRS morphology type (rows), while the mean value and standard deviation are calculated for all DTT values and noise levels. This table represents the detection quality and its independence from the QRS morphology, which is unknown at the time of detection.

Table 3 presents statistics on TP/TB for each algorithm (columns) and each noise level tested (rows), while the mean value and standard deviation are calculated for all QRS morphology types and DTT values. This table helps to explore which algorithm is the best and which gives the most stable detection results in the presence of noise.

### 4.2. Plots of the Detectors’ Performance

To provide deeper insight into detectors’ performance, we also use plots (displayed in Figure 7, Figure 8, Figure 9 and Figure 10) that examine the properties of each studied algorithm independently.

## 5. Discussion

### 5.1. Influence of Noise

While planning the experimental work, the expectation was that, with increasing levels of noise the TP/TB results would deteriorate. When we analyze the average TP/TB results in Table 3, we can conclude that the expectations are fulfilled for Algorithm 1, Algorithm 2, and Algorithm 3 but not for Algorithm 4.

The average TP/TB (Table 3) results for increased levels of noise are in the following range for no added noise to maximum added noise SNR = 3 dB, respectively:

- Algorithm 1—83.72% and 82.12%,
- Algorithm 2—90.68% and 89.18%,
- Algorithm 3—77.12% and 71.74%,
- and Algorithm 4—62.03% and 70.43%; there is no deterioration, but an improvement of 8.4% with added noise.

For Algorithm 1, the improvement in average TP/TB with increased level of added noise has not been observed (Table 3). Turning the analysis of Algorithm 1 to not-averaged TP/TB data (Figure 7), we can observe that, for DTT 8.33 ms (three samples) QRS morphology types N, L, R, and V, there is an improvement in TP/TB with increased added noise level for several records. Looking into details (Table 4) for QRS morphology of N type, DTT 8.33 ms (three samples), the TP/TB results for added noise 15 dB, 7 dB, and 3 dB are improved compared with records with no added noise for the following records: 103, 106, 112, 113, 115, 117, 119, 122, 123, 200, 201, 202, 203, 208, 210, 213, 219, 220, 221, 233, and 234 (Table 4). It constitutes results for over 50% of the records (21 from 40 records with N-type QRS morphology). The biggest improvement is registered for record 122. For greater values of DTT and N-type beats with this algorithm, there are no improvements in TP/TB (Figure 7); detailed results for DTT 47.22 ms (17 samples) are shown in Table 5.

For Algorithm 2, an improvement in average TP/TB with an increased level of added noise has not been observed (Table 3). As can be seen in the detailed data for N-type QRS morphology and DTT 8.33 ms (three samples) in Table 6, there is only one case of TP/TB improvement, for record 230. The improvement in TP results for no added noise versus added noise of 15, 7, and 3 dB are 86, 156, and 196, respectively.

For Algorithm 3, the improvement in average TP/TB with an increased level of added noise has not been observed (Table 3). When analyzing Algorithm 3 results for not-averaged TP/TB data (Figure 9), the improvement of TP/TB with an increased level of added noise is revealed for QRS morphology type V for all values of DTT. Analysis of the results for N-type QRS morphology (Table 8 and Table 9) reveals that, for DTT 8.33 ms (three samples), there is improvement in totals, whereas for DTT 47.22 ms (17 samples), there is no improvement in totals (only in a few selected records).

For Algorithm 4, the improvement in average TP/TB with an increased level of added noise has been observed (Table 3). Looking into details (Table 10) for QRS morphology of N-type, DTT 8.33 ms (three samples), the TP/TB results for added noise 15 dB, 7 dB, and 3 dB are improved for 35 out of 40 records. The improvement is not revealed for records 105, 119, 121, 122, and 212. For DTT 47.22 ms (17 samples), the improvement is revealed for 30 out of 40 records. The improvement is not revealed for the following records: 100, 105, 106, 119, 121, 122, 212, 222, 228, and 231.

Why do TP/TB results for Algorithm 4 not deteriorate with increased levels of noise? When we look into detailed data in Table 10, TP/TB results for N-type QRS morphology, and DTT 8.33 ms (three samples), the improvement in TP/TB with increasing levels of noise can be observed for 30 out of 40 records. Similar improvements of results are revealed for DTT 47.22 ms (17 samples) and can be observed in Table 11. Why do TP/TB results improve with added noise? Our hypothesis is that it is related to Algorithm 4 higher computational complexity being reflected in more calculations and processing blocks in computation. Thus, the hypothesis is that adding noise to the input ECG signal reduces friction between the blocks of computation. The phenomena of reduction of error from sticky moving parts in mechanical computers used to perform navigation and bomb trajectory calculations are cited as the first observation and purposeful use of dither [36]. In analog-to-digital conversion, dither—purposeful distortion to the input signal—causes digitization error to behave well statistically (dithered quantization) [7,8].

In summary, by adding muscular noise, we expected a deterioration of the detectors’ performance. Surprisingly, our results show that this is not always the case. For Algorithm 4, there is general improvement visible in averaged TP/TB data and confirmed in detailed data. For other algorithms, general improvement in averaged TP/TB has not been observed. Still, the phenomena of TP/TB improvement with added noise are present, although on a smaller scale for other algorithms (namely for specific QRS morphologies, DTT values, or individual records).

### 5.2. Influence of Detector Time Tolerance DTT

While planning the experimental work, the expectation was that increasing DTT would improve the *TP/TB* results. This assumption was additionally supported by results of one-dimensional analysis of *DTT* influence on *TP* in [10].

When we analyze the results in Table 1, we can see that results confirm the expectation for all algorithms and all DTT values under analysis. The average *TP/TB* results calculated over all added noise and QRS morphologies achieved for the lowest *DTT* 8.33 ms (three samples) and highest DTT 163.89 ms (59 samples), respectively, are:

- Algorithm 1—27.54% and 99.25%,
- Algorithm 2—65.75% and 99.71%,
- Algorithm 3—8.26% and 94.80%,
- and Algorithm 4—14.30% and 99.46%.

The following are additional observations from the analysis of plots (Figure 7, Figure 8, Figure 9 and Figure 10) for all algorithms:

- Algorithm 1 for QRS morphologies N, L, R, P, and A, with the exception of DTT 8.33 ms (three samples), reveals TP/TB of more than 90%. For QRS morphology type V, the TP/TB results are below 90% for all DTT ≤ 125 ms (45 samples). For DTT 8.33 ms (three samples), for all QRS morphologies, the TP/TB results are below 30%.
- Algorithm 2 results of TP/TB for all DTT and for QRS morphology N and A are above 80%. For L-, R-, V- and P-type QRS morphologies, the TP/TB results are above 90% for DTT > 86.11 ms (31 samples).
- Algorithm 3 TP/TB results for DTT 8.33 ms and for all QRS morphologies are below 30%, and for other higher DTT values, there is a clear deterioration of TP/TB results with decreasing DTT. For all QRS morphologies TP/TB is above 90% only for DTT 125 ms and 163.89 ms (45 and 59 samples).
- Algorithm 4 TP/TB results for all QRS morphologies are above 90% only for DTT 125 ms and 163.89 ms (45 and 59 samples). Results for other values of DTT clearly deteriorate well below 80% with decreasing DTT value.

In summary, decreasing DTT causes deterioration of TP/TB with varying ratios dependent on algorithm, QRS morphology, and added noise level.

### 5.3. Influence of QRS Morphology

While planning the experimental work, the expected result was that, depending on the algorithm, certain QRS morphologies are more difficult for precise R-peak detection than other QRS morphologies. During the analysis of average TP/TB results in Table 2, we observed that the aforementioned expectation is fulfilled for all algorithms.

On closer analysis of the range of TP/TB results from worst to best in Table 2, we can observe the following range of TP/TB results for algorithm and QRS morphology, respectively:

- Algorithm 1 from 76.92% for V to 92.52% for P,
- Algorithm 2 from 80.51% for V to 97.24% for A,
- Algorithm 3 from 67.29% for V to 76.47% for L,
- and Algorithm 4 from 65.38% for L to 72.28% for N.

We can conclude that, for the four algorithms under analysis, the most difficult in precise R-peak location was V-type QRS morphology for three algorithms and L-type for one algorithm. In terms of the easiest QRS morphology type for precise R-peak location, it is different for each algorithm under analysis and yields P-, A-, L-, and N-types of QRS morphology.

Visual analysis of the TP/TB results displayed in plots (Figure 7, Figure 8, Figure 9 and Figure 10) in a direction to reveal any visual similarities in locations of the data points for various QRS morphologies is presented below for algorithm and QRS morphology, respectively:

- Algorithm 1 for N, R, and A—similar locations; for L, V, and P, each type—different locations,
- Algorithm 2 for N, R, and A—similar locations; for L, V, and P—similar locations,
- Algorithm 3 for N, L, and R—similar locations; for V, P, and A, each type—different locations,
- and Algorithm 4 for N, R, and A—similar locations; for L, V, and P—similar locations.

In summary, the most difficult and easiest QRS morphology type for accurate R-peak detection by a given detection algorithm is specific for that algorithm. The worst and best TP/TB results for all levels of noise in ECG signal and all DTT values under analysis are also specific for each algorithm.

### 5.4. Comparison of the Algorithms Studied

It is evident that a high-performance QRS detector should demonstrate a high TP/TB ratio even with low DTT. Therefore, a series of QRS detection instants may reliably be used by following procedures such as heart rate variability. In this aspect, all atrial premature, blocked, paced, and ventricular beats are not considered as representative to cardiac cycle control from the autonomous nervous system. Consequently, the precision of N-type QRS detection is more important than other types of beat morphology. At the same time, the high stability of the detection point sequence (that is, its independence from QRS morphology) is a desired algorithm feature. Lastly, an algorithm’s detection robustness to noise is very important, especially for mobile and wearable applications, as the level of noise is high and fluctuates depending on the environment and human activity.

Algorithm 1 and Algorithm 2 demonstrate good robustness to noise in ECG signal (Table 3, Figure 7 and Figure 8), whereas the QRS detection accuracy for Algorithm 3 and Algorithm 4 for noisy ECG signal is significantly worse. Additionally, the detection accuracy strongly drops with noise level, which makes the Algorithm 3 and Algorithm 4 not suitable for implementation in wearable ECG devices.

Algorithm 2 has a good detection accuracy even for low temporal tolerances (DTT = 3), except for V-type beats it has a TP/TB score over 50%, and for DTT = 17, it grows over 93% except for types L and V. This is a clinically acceptable detection ratio for remote cardiac rhythm detection and, as our results show, can be achieved with a temporal accuracy of 47.22 ms (17 samples).

Algorithm 1 and Algorithm 3 work well for DTT ≥ 47.22 ms (17 samples); TP/TB exceeds 90%, except for QRS types V for Algorithm 1 and Algorithm 3, and P for Algorithm 3, but its performance drops for low temporal tolerance (DTT 8.33 ms, three samples). Compared with Algorithm 1, Algorithm 2, and Algorithm 3, which were developed in the last decade, Algorithm 4, developed in 1985 and broadly referenced in the literature on QRS detection, is more sensitive to noise (Figure 10) and QRS morphology. It achieves satisfactory detection accuracy only for large DTT values.

Algorithm 1 (Figure 7) shows perfect noise immunity for V-type QRS morphology and high noise immunity for other beat types. For low noise levels, it has similar scores to Algorithm 3 (Figure 9), except for P morphology, where it is, again, significantly better.

Adding noise improves the results of Algorithm 3 in V-type morphology, and for high DTT also in N-type QRS morphology. This is also observed in Algorithm 4 (Figure 10), where, in most cases, red dots (SNR = 3 dB) are not the lowest data points in the plot.

### 5.5. Limitations of the Study and Future Research

A lack of computational complexity analysis may be considered as the main limitation of our study. Although the number of elementary operations for each algorithm and the growth of resource demand related to input size can be easily determined, preferences in this aspect depend on the target platform. The hardware architecture (e.g., multicore processors, vector data processing) and machine representation of data determine principal usability factors, such as maximum time of autonomy or the necessary size of battery, particularly in wearable recorders.

Another limitation of our study is the use of the MIT-DIH Arrhythmia Database, which is relatively sparsely sampled (sampling frequency 360 Hz) and known for annotation errors. In fact, the position of the maximum of the signal depends on the ECG lead used (each lead “sees” the spatial electrical phenomenon of heartbeat from a different viewpoint) and the true QRS maximum (i.e., precise R-peak) position can only be estimated from vectorcardiography. Moreover, in all cases, the sampling process is in no way synchronized with heart action. Consequently, the maximum falls between samples, and calculation of its true position requires interpolation. Nevertheless, we assume that possible annotation errors are rare and equally distributed before and after the true positions of R-peaks. This may result in an overestimation of the standard deviation results, but with no effect on the mean results of the algorithms.

The above two limitations will be addressed in future research, as will be the analysis of the sources of improvement in TP/TB results under conditions of added noise, as discussed in Section 5.1.

## 6. Conclusions

This study focused on the performance evaluation of QRS detectors aware of temporal accuracy and the presence of noise. Contrarily to most authors, satisfied with detection correctness as the sole quality indicator, we propose multidimensional criteria, including

- accuracy (i.e., TP/TB),
- precision of detection point location, i.e., TP/TB dependence on the DTT,
- sensitivity to noise,
- and sensitivity to QRS morphology.

To show the multidimensional method for QRS detectors’ evaluation of temporal accuracy, we compared three modern QRS detection algorithms and a well-known, commonly referenced Pan–Tompkins algorithm originating from the pioneer age of computerized electrocardiography. 

The main scientific contribution of this work lies in testing QRS detectors in multiple dimensions, including various time tolerance values (DTT), which determines the detection statistics expressed by true positive, false positive, and false negative detection cases. Algorithms that demonstrate good accuracy with low values of DTT are the most precise algorithms. Output from such algorithms is immediately usable for subsequent ECG processing procedures such as HRV analysis or QRS morphology classification. Moreover, we observed that the accuracy of QRS detection depends on QRS morphology. Tests performed for various levels of added muscular noise revealed that there are significant differences between algorithms with respect to their noise robustness. High robustness amid noise (stable detection accuracy in noisy ECG recordings) is important in mobile and wearable applications in unstable conditions (e.g., in motion).

## Figures and Tables

**Table 1:** QRS detectors’ accuracy is expressed as the true-positive-to-total-beats ratio for various DTT values. The mean value and the standard deviation of the true-positive-to-total-beats ratio are computed for all six considered QRS morphology types and all four levels of added noise. The best results are highlighted in bold.

Algorithm Detector Temporal Tolerance [ms] (Samples)	Algorithm 1		Algorithm 2		Algorithm 3		Algorithm 4	
	Mean	Std	Mean	Std	Mean	Std	Mean	Std
8.33 (3)	27.54	21.93	**65.75**	19.74	8.26	9.52	14.30	6.36
47.22 (17)	**92.53**	9.27	87.77	11.16	80.66	11.68	52.85	14.17
86.11 (31)	96.79	4.44	**98.66**	1.62	88.43	6.25	74.93	8.83
125.00 (45)	98.89	1.02	**99.55**	0.44	92.22	4.72	98.52	1.33
163.89 (59)	99.25	0.89	**99.71**	0.36	94.80	4.45	99.46	0.55

**Table 2:** QRS detectors’ accuracy is expressed as the true-positive-to-total-beats ratio for various heartbeat morphology types. The mean value and the standard deviation of the true-positive-to-total-beats ratio are computed for all five considered DTT values and all four levels of added noise. The best results are highlighted in bold.

Algorithm Beat Type	Algorithm 1		Algorithm 2		Algorithm 3		Algorithm 4	
	Mean	Std	Mean	Std	Mean	Std	Mean	Std
N	82.71	32.34	**96.30**	5.66	74.12	36.91	72.28	30.12
L	82.47	31.34	**86.86**	19.30	76.47	35.31	65.38	36.50
R	80.96	36.85	**93.22**	11.94	75.52	37.60	71.27	30.76
V	76.92	26.34	**80.51**	24.54	67.29	34.08	66.36	34.20
P	**92.52**	10.41	85.94	18.55	72.68	25.64	66.49	33.43
A	82.42	35.74	**97.24**	5.22	71.17	34.75	66.30	35.91

**Table 3:** QRS detectors’ accuracy is expressed as the true-positive-to-total-beats ratio for various added noise levels. The mean value and the standard deviation of the true-positive-to-total-beats ratio are computed for all six considered QRS morphology types and all five considered DTT values. The best results are highlighted in bold.

Algorithm Added Noise Level	Algorithm 1		Algorithm 2		Algorithm 3		Algorithm 4	
	Mean	Std	Mean	Std	Mean	Std	Mean	Std
No noise added	83.72	30.88	**90.68**	16.80	77.12	35.90	62.03	36.60
SNR 15 dB	83.38	30.48	**90.34**	16.78	72.16	33.62	69.27	32.71
SNR 7 dB	82.78	30.08	**89.86**	16.93	70.47	33.00	70.43	31.67
SNR 3 dB	82.12	29.71	**89.18**	17.20	71.74	33.50	70.32	31.60

**Table 4:** Results for Algorithm 1, N-type morphology beats and DTT = 3 (8.33 ms), from the MIT-BIH AD. Improvements in results are marked in bold. Records 107, 109, 111, 118, 124, 207, 214, and 232 are not shown, as there are no N-type morphology beats in those records.

MIT-BIH Arrhythmia Database, Normal—N-Type Beats		No Noise Added	SNR = 15 dB	SNR = 7 dB	SNR = 3 dB	SNR = 15 dB vs. No Noise Added	SNR = 7 dB vs. No Noise Added	SNR = 3 dB vs. No Noise Added
Record	TB	TP	TP	TP	TP			
100	2239	1337	1176	1143	1123	−161	−194	−214
101	1860	1860	1853	1806	1766	−7	−54	−94
102	99	99	92	86	80	−7	−13	−19
103	2082	114	261	334	374	**147**	**220**	**260**
104	163	1	1	4	6	0	**3**	**5**
105	2526	2383	2305	2203	2128	−78	−180	−255
106	1507	47	122	158	182	**75**	**111**	**135**
108	1739	660	552	518	512	−108	−142	−148
112	2537	0	93	126	137	**93**	**126**	**137**
113	1789	0	29	117	148	**29**	**117**	**148**
114	1820	521	456	440	421	−65	−81	−100
115	1953	0	9	32	55	**9**	**32**	**55**
116	2302	1	51	139	178	**50**	**138**	**177**
117	1534	619	565	520	489	−54	−99	−130
119	1543	12	278	330	360	**266**	**318**	**348**
121	1861	1165	894	760	679	−271	−405	−486
122	2476	254	664	751	777	**410**	**497**	**523**
123	1515	3	77	59	79	**74**	**56**	**76**
200	1743	1	10	34	61	**9**	**33**	**60**
201	1625	16	66	100	137	**50**	**84**	**121**
202	2061	3	91	136	156	**88**	**133**	**153**
203	2529	280	320	360	371	**40**	**80**	**91**
205	2571	1394	1346	1290	1263	−48	−104	−131
208	1586	164	288	360	386	**124**	**196**	**222**
209	2621	1	1	4	10	0	**3**	**9**
210	2423	16	101	186	234	**85**	**170**	**218**
212	923	6	28	55	78	**22**	**49**	**72**
213	2641	8	33	101	149	**25**	**93**	**141**
215	3195	2	3	3	4	**1**	**1**	**2**
217	244	0	2	8	11	**2**	**8**	**11**
219	2082	8	111	210	250	**103**	**202**	**242**
220	1954	2	7	36	44	**5**	**34**	**42**
221	2031	0	12	39	80	**12**	**39**	**80**
222	2062	814	841	832	822	**27**	**18**	**8**
223	2029	0	9	40	77	**9**	**40**	**77**
228	1688	1204	1094	1015	972	−110	−189	−232
230	2255	0	0	2	3	0	**2**	**3**
231	314	265	219	226	229	−46	−39	−36
233	2230	191	346	431	470	**155**	**240**	**279**
234	2700	11	215	344	413	**204**	**333**	**402**
TOTAL	75,052	13,462	14,621	15,338	15,714	**1159**	**1876**	**2252**

**Table 5:** Results for Algorithm 1, N-type morphology beats and DTT = 17 (47.22 ms), from the MIT-BIH AD. Improvements in results are marked in bold. Records 107, 109, 111, 118, 124, 207, 214, and 232 are not shown, as there are no N-type morphology beats in those records.

MIT-BIH Arrhythmia Database, Normal—N-Type Beats		No Noise Added	SNR = 15 dB	SNR = 7 dB	SNR = 3 dB	SNR = 15 dB vs. No Noise Added	SNR = 7 dB vs. No Noise Added	SNR = 3 dB vs. No Noise Added
Record	TB	TP	TP	TP	TP			
100	2239	2238	2238	2232	2224	0	−6	−14
101	1860	1860	1856	1820	1797	−4	−40	−63
102	99	99	98	95	92	−1	−4	−7
103	2082	2082	2082	2081	2077	0	−1	−5
104	163	163	163	163	161	0	0	−2
105	2526	2508	2510	2502	2477	**2**	−6	−31
106	1507	1504	1467	1425	1398	−37	−79	−106
108	1739	1666	1527	1459	1459	−139	−207	−207
112	2537	2536	2487	2343	2220	−49	−193	−316
113	1789	1788	1779	1770	1752	−9	−18	−36
114	1820	1778	1721	1659	1606	−57	−119	−172
115	1953	1952	1952	1930	1920	0	−22	−32
116	2302	2284	2280	2262	2232	−4	−22	−52
117	1534	1534	1437	1360	1319	−97	−174	−215
119	1543	1543	1490	1454	1428	−53	−89	−115
121	1861	1860	1694	1532	1403	−166	−328	−457
122	2476	2476	2471	2434	2376	−5	−42	−100
123	1515	1515	1462	1447	1437	−53	−68	−78
200	1743	1740	1738	1730	1725	−2	−10	−15
201	1625	1610	1595	1588	1586	−15	−22	−24
202	2061	2061	2037	2028	2021	−24	−33	−40
203	2529	2471	2455	2409	2382	−16	−62	−89
205	2571	2570	2570	2568	2563	0	−2	−7
208	1586	1579	1576	1572	1565	−3	−7	−14
209	2621	2621	2621	2621	2621	0	0	0
210	2423	2421	2417	2415	2405	−4	−6	−16
212	923	922	922	922	922	0	0	0
213	2641	2640	2640	2637	2637	0	−3	−3
215	3195	3194	3194	3194	3194	0	0	0
217	244	244	244	243	239	0	−1	−5
219	2082	2082	2069	2039	2032	−13	−43	−50
220	1954	1954	1954	1951	1929	0	−3	−25
221	2031	2031	2028	2020	2011	−3	−11	−20
222	2062	2048	2022	1983	1962	−26	−65	−86
223	2029	2029	2027	2019	2005	−2	−10	−24
228	1688	1643	1557	1508	1481	−86	−135	−162
230	2255	2255	2255	2254	2253	0	−1	−2
231	314	289	255	260	264	−34	−29	−25
233	2230	2228	2229	2226	2217	**1**	−2	−11
234	2700	2697	2699	2699	2699	**2**	**2**	**2**
TOTAL	75,052	74,715	73,818	72,854	72,091	−897	−1861	−2624

**Table 6:** Results for Algorithm 2, N-type morphology beats and DTT = 3 (8.33 ms), from the MIT-BIH AD. Improvements in results are marked in bold. Records 107, 109, 111, 118, 124, 207, 214, and 232 are not shown, as there are no N-type beats in those records.

MIT-BIH Arrhythmia Database, Normal—N-Type Beats		No Noise Added	SNR = 15 dB	SNR = 7 d	SNR = 3 dB	SNR = 15 dB vs. No Noise Added	SNR = 7 dB vs. No Noise Added	SNR = 3 dB vs. No Noise Added
Record	TB	TP	TP	TP	TP			
100	2239	2236	2235	2229	2198	−1	−7	−38
101	1860	1856	1855	1856	1849	−1	0	−7
102	99	92	84	75	67	−8	−17	−25
103	2082	2080	2080	2079	2077	0	−1	−3
104	163	163	163	161	158	0	−2	−5
105	2526	2465	2444	2362	2267	−21	−103	−198
106	1507	1489	1489	1478	1471	0	−11	−18
108	1739	705	652	641	644	−53	−64	−61
112	2537	1637	1458	1266	1150	−179	−371	−487
113	1789	1787	1788	1788	1788	**1**	**1**	**1**
114	1820	521	505	475	455	−16	−46	−66
115	1953	1948	1947	1934	1916	−1	−14	−32
116	2302	2268	2222	2128	2048	−46	−140	−220
117	1534	1002	852	753	710	−150	−249	−292
119	1543	1542	1530	1455	1398	−12	−87	−144
121	1861	1609	1151	1002	903	−458	−607	−706
122	2476	2475	2316	2130	2014	−159	−345	−461
123	1515	1513	1494	1431	1362	−19	−82	−151
200	1743	1623	1575	1509	1446	−48	−114	−177
201	1625	1622	1622	1603	1575	0	−19	−47
202	2061	2061	2058	2038	1991	−3	−23	−70
203	2529	1810	1618	1497	1390	−192	−313	−420
205	2571	2567	2567	2549	2518	0	−18	−49
208	1586	1569	1568	1557	1529	−1	−12	−40
209	2621	2616	2616	2604	2586	0	−12	−30
210	2423	2417	2408	2355	2292	−9	−62	−125
212	923	922	922	921	920	0	−1	−2
213	2641	2637	2637	2616	2575	0	−21	−62
215	3195	1997	1960	1915	1901	−37	−82	−96
217	244	238	231	223	216	−7	−15	−22
219	2082	2077	2069	1992	1936	−8	−85	−141
220	1954	1939	1884	1852	1799	−55	−87	−140
221	2031	2030	2031	2020	2000	**1**	−10	−30
222	2062	2049	2037	2012	1964	−12	−37	−85
223	2029	1910	1873	1785	1709	−37	−125	−201
228	1688	1675	1672	1616	1560	−3	−59	−115
230	2255	215	301	371	411	**86**	**156**	**196**
231	314	314	314	314	314	0	0	0
233	2230	2223	2210	2135	2083	−13	−88	−140
234	2700	2696	2697	2696	2684	**1**	0	−12
TOTAL	75,052	66,595	65,135	63,423	61,874	−1460	−3172	−4721

**Table 7:** Results for Algorithm 2, N-type morphology beats and DTT = 17 (47.22 ms), from the MIT-BIH AD. Records 107, 109, 111, 118, 124, 207, 214, and 232 are not shown, as there are no N-type beats in those records.

MIT-BIH Arrhythmia Database, Normal—N-Type Beats		No Noise Added	SNR = 15 dB	SNR = 7 dB	SNR = 3 dB	SNR = 15 dB vs. No Noise Added	SNR = 7 dB vs. No Noise Added	SNR = 3 dB vs. No Noise Added
Record	TB	TP	TP	TP	TP			
100	2239	2236	2235	2232	2214	−1	−4	−22
101	1860	1856	1855	1856	1852	−1	0	−4
102	99	95	92	90	82	−3	−5	−13
103	2082	2080	2080	2080	2079	0	0	−1
104	163	163	163	163	162	0	0	−1
105	2526	2484	2479	2427	2378	−5	−57	−106
106	1507	1490	1492	1486	1485	2	−4	−5
108	1739	1610	1574	1556	1547	−36	−54	−63
112	2537	2536	2432	2270	2177	−104	−266	−359
113	1789	1787	1788	1788	1788	1	1	1
114	1820	1817	1811	1769	1723	−6	−48	−94
115	1953	1950	1950	1948	1938	0	−2	−12
116	2302	2275	2268	2228	2195	−7	−47	−80
117	1534	1534	1446	1381	1328	−88	−153	−206
119	1543	1542	1533	1480	1434	−9	−62	−108
121	1861	1860	1682	1579	1485	−178	−281	−375
122	2476	2475	2424	2339	2289	−51	−136	−186
123	1515	1513	1513	1491	1462	0	−22	−51
200	1743	1741	1739	1729	1716	−2	−12	−25
201	1625	1623	1623	1615	1604	0	−8	−19
202	2061	2061	2061	2049	2027	0	−12	−34
203	2529	2449	2344	2262	2188	−105	−187	−261
205	2571	2567	2567	2552	2525	0	−15	−42
208	1586	1569	1568	1563	1548	−1	−6	−21
209	2621	2620	2619	2618	2617	−1	−2	−3
210	2423	2418	2415	2394	2365	−3	−24	−53
212	923	922	922	921	921	0	−1	−1
213	2641	2638	2639	2634	2608	1	−4	−30
215	3195	3194	3194	3191	3187	0	−3	−7
217	244	244	243	242	240	−1	−2	−4
219	2082	2079	2077	2044	2007	−2	−35	−72
220	1954	1953	1951	1948	1939	−2	−5	−14
221	2031	2030	2031	2027	2015	1	−3	−15
222	2062	2055	2044	2021	1983	−11	−34	−72
223	2029	2028	2024	1999	1967	−4	−29	−61
228	1688	1677	1677	1635	1605	0	−42	−72
230	2255	2252	2252	2252	2252	0	0	0
231	314	314	314	314	314	0	0	0
233	2230	2224	2224	2190	2162	0	−34	−62
234	2700	2696	2697	2698	2689	1	2	−7
TOTAL	75,052	74,657	74,042	73,061	72,097	−615	−1596	−2560

**Table 8:** Results for Algorithm 3, N-type morphology beats and DTT = 3 (8.33 ms), from the MIT-BIH AD. Improvements in results are marked in bold. Records 107, 109, 111, 118, 124, 207, 214, and 232 are not shown, as there are no N-type morphology beats in those records.

MIT-BIH Arrhythmia Database, Normal—N-Type Beats		No Noise Added	SNR = 15 dB	SNR = 7 dB	SNR = 3 dB	SNR = 15 dB vs. No Noise Added	SNR = 7 dB vs. No Noise Added	SNR = 3 dB vs. No Noise Added
Record	TB	TP	TP	TP	TP			
100	2239	202	197	168	156	−5	−34	−46
101	1860	16	32	32	36	**16**	**16**	**20**
102	99	0	0	1	1	0	**1**	**1**
103	2082	2	3	2	1	**1**	0	−1
104	163	8	9	12	8	**1**	**4**	0
105	2526	35	37	45	56	**2**	**10**	**21**
106	1507	0	1	0	2	**1**	0	**2**
108	1739	2	6	17	28	**4**	**15**	**26**
112	2537	58	165	208	239	**107**	**150**	**181**
113	1789	0	0	0	0	0	0	0
114	1820	244	224	228	211	−20	−16	−33
115	1953	0	0	0	1	0	0	**1**
116	2302	0	2	3	8	**2**	**3**	**8**
117	1534	679	530	490	488	−149	−189	−191
119	1543	0	0	1	1	0	**1**	**1**
121	1861	0	32	66	89	**32**	**66**	**89**
122	2476	0	1	7	11	**1**	**7**	**11**
123	1515	0	0	0	0	0	0	0
200	1743	0	0	0	10	0	0	**10**
201	1625	0	2	4	5	**2**	**4**	**5**
202	2061	0	1	3	2	**1**	**3**	**2**
203	2529	39	62	82	101	**23**	**43**	**62**
205	2571	29	37	42	47	**8**	**13**	**18**
208	1586	89	90	79	80	**1**	−10	−9
209	2621	2	1	5	7	−1	**3**	**5**
210	2423	0	7	12	15	**7**	**12**	**15**
212	923	0	0	1	1	0	**1**	**1**
213	2641	0	0	2	1	0	**2**	**1**
215	3195	1	4	12	17	**3**	**11**	**16**
217	244	0	0	0	1	0	0	**1**
219	2082	2	2	2	4	0	0	**2**
220	1954	0	1	1	1	**1**	**1**	**1**
221	2031	0	1	2	4	**1**	**2**	**4**
222	2062	630	662	607	587	**32**	−23	−43
223	2029	0	1	6	6	**1**	**6**	**6**
228	1688	6	20	27	32	**14**	**21**	**26**
230	2255	0	0	0	1	0	0	**1**
231	314	0	0	0	0	0	0	0
233	2230	0	0	2	2	0	**2**	**2**
234	2700	0	0	0	1	0	0	**1**
TOTAL	75,052	2044	2130	2169	2261	**86**	**125**	**217**

**Table 9:** Results for Algorithm 3, N-type morphology beats and DTT = 17 (47.22 ms), from the MIT-BIH AD. Improvements in results are marked in bold. Records 107, 109, 111, 118, 124, 207, 214, and 232 are not shown, as there are no N-type morphology beats in those records.

MIT-BIH Arrhythmia Database, Normal—N-TYPE beats		No Noise Added	SNR = 15 dB	SNR = 7 dB	SNR = 3 dB	SNR = 15 dB vs. No Noise Added	SNR = 7 dB vs. No Noise Added	SNR = 3 dB vs. No Noise Added
Record	TB	TP	TP	TP	TP			
100	2239	2239	2104	1906	1829	−135	−333	−410
101	1860	1853	1697	1530	1452	−156	−323	−401
102	99	99	88	82	77	−11	−17	−22
103	2082	2082	1954	1759	1690	−128	−323	−392
104	163	159	156	146	138	−3	−13	−21
105	2526	2440	2166	1999	1987	−274	−441	−453
106	1507	1500	1331	1227	1202	−169	−273	−298
108	1739	1570	1256	1149	1160	−314	−421	−410
112	2537	2534	2101	2079	2014	−433	−455	−520
113	1789	1789	1684	1556	1539	−105	−233	−250
114	1820	1393	1432	1356	1277	**39**	−37	−116
115	1953	1953	1618	1535	1545	−335	−418	−408
116	2302	2277	1859	1884	1950	−418	−393	−327
117	1534	1532	1236	1208	1242	−296	−324	−290
119	1543	1543	1168	1194	1225	−375	−349	−318
121	1861	1857	1436	1375	1345	−421	−482	−512
122	2476	2474	1948	1991	2012	−526	−483	−462
123	1515	1515	1158	1147	1164	−357	−368	−351
200	1743	1711	0	0	1408	−1711	−1711	−303
201	1625	1621	1609	1497	1406	−12	−124	−215
202	2061	2058	1974	1799	1723	−84	−259	−335
203	2529	2453	2236	2078	2042	−217	−375	−411
205	2571	2567	2456	2239	2174	−111	−328	−393
208	1586	1559	1429	1325	1307	−130	−234	−252
209	2621	2607	2562	2389	2329	−45	−218	−278
210	2423	2393	2350	2164	2004	−43	−229	−389
212	923	916	862	799	746	−54	−117	−170
213	2641	2632	2346	2221	2208	−286	−411	−424
215	3195	3187	3156	3003	2916	−31	−184	−271
217	244	244	217	198	199	−27	−46	−45
219	2082	2082	1724	1671	1659	−358	−411	−423
220	1954	1953	1642	1534	1537	−311	−419	−416
221	2031	2027	1955	1754	1726	−72	−273	−301
222	2062	1665	1712	1613	1530	**47**	−52	−135
223	2029	2028	1740	1670	1619	−288	−358	−409
228	1688	1649	1540	1381	1336	−109	−268	−313
230	2255	2245	2089	1904	1831	−156	−341	−414
231	314	314	296	254	246	−18	−60	−68
233	2230	2228	1980	1836	1839	−248	−392	−389
234	2700	2699	2540	2346	2251	−159	−353	−448
TOTAL	75,052	73,647	64,807	60,798	60,884	−8840	−12,849	−12,763

**Table 10:** Results for Algorithm 4, N-type morphology beats and DTT = 3 (8.33 ms), from the MIT-BIH AD. Improvements in results are marked in bold. Records 107, 109, 111, 118, 124, 207, 214, and 232 are not shown, as there are no N-type morphology beats in those records.

MIT-BIH Arrhythmia Database, Normal—N-Type Beats		No Noise Added	SNR = 15 dB	SNR = 7 dB	SNR = 3 dB	SNR = 15 dB vs. No Noise Added	SNR = 7 dB vs. No Noise Added	SNR = 3 dB vs. No Noise Added
Record	TB	TP	TP	TP	TP			
100	2239	376	522	608	616	**146**	**232**	**240**
101	1860	303	434	500	516	**131**	**197**	**213**
102	99	16	31	23	21	**15**	**7**	**5**
103	2082	197	301	411	448	**104**	**214**	**251**
104	163	18	23	29	29	**5**	**11**	**11**
105	2526	2178	2021	1778	1626	−157	−400	−552
106	1507	396	432	476	480	**36**	**80**	**84**
108	1739	96	209	232	223	**113**	**136**	**127**
112	2537	7	155	131	105	**148**	**124**	**98**
113	1789	199	289	370	415	**90**	**171**	**216**
114	1820	71	138	137	132	**67**	**66**	**61**
115	1953	4	84	157	196	**80**	**153**	**192**
116	2302	64	337	365	385	**273**	**301**	**321**
117	1534	142	308	313	292	**166**	**171**	**150**
119	1543	756	796	667	588	**40**	−89	−168
121	1861	663	467	153	87	−196	−510	−576
122	2476	2123	1734	1383	1141	−389	−740	−982
123	1515	7	74	147	153	**67**	**140**	**146**
200	1743	1	20	52	70	**19**	**51**	**69**
201	1625	27	117	185	228	**90**	**158**	**201**
202	2061	11	48	127	204	**37**	**116**	**193**
203	2529	114	210	221	227	**96**	**107**	**113**
205	2571	561	741	788	730	**180**	**227**	**169**
208	1586	314	440	441	408	**126**	**127**	**94**
209	2621	71	186	304	355	**115**	**233**	**284**
210	2423	54	329	400	464	**275**	**346**	**410**
212	923	514	511	499	490	−3	−15	−24
213	2641	772	1714	1635	1392	**942**	**863**	**620**
215	3195	3	24	90	129	**21**	**87**	**126**
217	244	0	14	19	22	**14**	**19**	**22**
219	2082	75	466	563	537	**391**	**488**	**462**
220	1954	7	74	161	196	**67**	**154**	**189**
221	2031	45	200	312	355	**155**	**267**	**310**
222	2062	543	607	600	593	**64**	**57**	**50**
223	2029	10	73	107	97	**63**	**97**	**87**
228	1688	336	543	504	504	**207**	**168**	**168**
230	2255	0	2	11	28	**2**	**11**	**28**
231	314	106	111	116	126	**5**	**10**	**20**
233	2230	14	209	321	337	**195**	**307**	**323**
234	2700	706	872	953	997	**166**	**247**	**291**
TOTAL	75,052	11,900	15,866	16,289	15,942	**3966**	**4389**	**4042**

**Table 11:** Results for Algorithm 4, N-type morphology beats and DTT = 17 (47.22 ms), from the MIT-BIH AD. Improvements in results are marked in bold. Records 107, 109, 111, 118, 124, 207, 214, and 232 are not shown, as there are no N-type morphology beats in those records.

MIT-BIH Arrhythmia Database, Normal—N-Type Beats		No Noise Added	SNR = 15 dB	SNR = 7 dB	SNR = 3 dB	SNR = 15 dB vs. No Noise Added	SNR = 7 dB vs. No Noise Added	SNR = 3 dB vs. No Noise Added
Record	TB	TP	TP	TP	TP			
100	2239	1920	1889	1909	1851	−31	−11	−69
101	1860	1332	1359	1381	1361	**27**	**49**	**29**
102	99	20	45	37	36	**25**	**17**	**16**
103	2082	1339	1480	1554	1602	**141**	**215**	**263**
104	163	58	85	96	93	**27**	**38**	**35**
105	2526	2372	2281	2093	2001	−91	−279	−371
106	1507	1500	1473	1438	1399	−27	−62	−101
108	1739	457	891	929	933	**434**	**472**	**476**
112	2537	1117	1503	1545	1462	**386**	**428**	**345**
113	1789	645	958	1107	1185	**313**	**462**	**540**
114	1820	579	873	920	923	**294**	**341**	**344**
115	1953	393	768	956	1017	**375**	**563**	**624**
116	2302	1388	1745	1744	1721	**357**	**356**	**333**
117	1534	409	857	907	905	**448**	**498**	**496**
119	1543	1508	1408	1266	1211	−100	−242	−297
121	1861	1036	1123	979	873	**87**	−57	−163
122	2476	2459	2205	2038	1958	−254	−421	−501
123	1515	313	736	864	859	**423**	**551**	**546**
200	1743	678	952	1040	1074	**274**	**362**	**396**
201	1625	964	1131	1184	1183	**167**	**220**	**219**
202	2061	895	1216	1333	1353	**321**	**438**	**458**
203	2529	1277	1649	1668	1692	**372**	**391**	**415**
205	2571	2018	2159	2137	2087	**141**	**119**	**69**
208	1586	1127	1271	1267	1216	**144**	**140**	**89**
209	2621	957	1354	1582	1656	**397**	**625**	**699**
210	2423	1026	1515	1622	1649	**489**	**596**	**623**
212	923	856	842	848	829	−14	−8	−27
213	2641	1558	2161	2180	2083	**603**	**622**	**525**
215	3195	922	1300	1521	1651	**378**	**599**	**729**
217	244	55	135	145	149	**80**	**90**	**94**
219	2082	726	1442	1536	1503	**716**	**810**	**777**
220	1954	1106	1277	1351	1372	**171**	**245**	**266**
221	2031	739	1235	1379	1438	**496**	**640**	**699**
222	2062	1870	1840	1795	1754	−30	−75	−116
223	2029	1220	1368	1359	1350	**148**	**139**	**130**
228	1688	1375	1406	1285	1242	**31**	−90	−133
230	2255	722	813	928	1009	**91**	**206**	**287**
231	314	291	278	278	283	−13	−13	−8
233	2230	1190	1458	1504	1483	**268**	**314**	**293**
234	2700	1943	2209	2270	2254	**266**	**327**	**311**
TOTAL	75,052	42,360	50,690	51,975	51,700	**8330**	**9615**	**9340**

## Data Availability

Not applicable, data used are already available as public database.
